# *In vitro* Analysis of Antioxidant, Antimicrobial and Antiproliferative Activity of *Enteromorpha antenna*, *Enteromorpha linza* and *Gracilaria corticata* Extracts

**DOI:** 10.17795/jjnpp-11277

**Published:** 2013-11-02

**Authors:** Manoj Kumar Narasimhan, Shenoy K Pavithra, Vishnupriya Krishnan, Muthukumaran Chandrasekaran

**Affiliations:** 1 Department of Genetic Engineering, School of Bio-engineering, SRM University, Kattankulathur, India; 2 Department of Industrial Biotechnology, Government College of Technology, Coimbatore, India

**Keywords:** Antioxidants, Reactive Oxygen Species, Seaweed

## Abstract

**Background:**

Seaweeds are taxonomically diverse benthic algae, which are rich in bioactive compounds. These compounds have a potential application in medicine.

**Objectives:**

The aim of the study was to investigate the bioactive properties of three seaweed samples, *Enteromorpha antenna*, *Enteromorpha linza* and *Gracilaria corticata* were collected from the shoreline of Mahabalipuram, Tamilnadu.

**Materials and Methods:**

Bioactive components were extracted by using various solvents. Antioxidant analysis methods like scavenging activity of nitric oxide, hydrogen peroxide, hydroxyl radicals, free radical scavenging (DPPH), FRAP (ferric reducing ability plasma) ability and reducing power were carried out. MTT assay was employed to study the anticancer activity against cancer cell lines Hep-G2, MCF7 and normal VERO cell lines.

**Results:**

It was found that methanolic extracts elicited higher total phenolic content, higher percentage scavenging activity of nitric oxide, hydrogen peroxide, hydroxyl radicals, free radical scavenging (DPPH), FRAP (ferric reducing ability plasma) ability and reducing power. Different concentrations of crude methanolic extracts of seaweeds showed potential antimicrobial activity by well diffusion method. Crude methanolic extract of *G. corticata* had significant anticancer activity followed by *E. antenna* and *E. linza* on cancer cell lines Hep-G2, MCF7 and normal VERO cell lines by MTT assay.

**Conclusions:**

The methanolic extracts of seaweeds *Enteromorpha antenna*, *Enteromorpha linza* and *Gracilaria corticata* possess high total phenolic content and shows a good free radical scavenging activity and hence are proven to have better antioxidant activity and they might be good candidates for further investigations in order to develop potential anticancer drugs.

## 1. Background

Seaweeds or marine algae are potentially prolific sources of high bioactive secondary metabolites that might represent useful leads in the development of pharmaceutical products. India’s coastline, which spreads along approximately 7500 kms, greatly differs in its geomorphological and hydrological characters at various regions. The sandy and rocky shallow sub tidal regions favor the growth of marine algae. The nutritional composition among the seaweeds depends on the environmental conditions and shore characteristics. Seaweeds are classified as *Rhodophyta* (red algae), *Phaeophyta* (brown algae) and *Chlorophyta* (green algae) depending on their nutrient and chemical composition. The growth of seaweeds favor in high light and oxygen concentration but in these conditions photo damaging and free radical production may result. Since the sea weeds possess anti-oxidative mechanism and compounds, they protect themselves from stress due to free radical formation and serious photodynamic damage (1). A number of factors influence the bioactive potential of seaweeds such as stage of fertility period, weather conditions and location. The crude extracts of most seaweeds show high bioactive potential during their fertility period (2). Antioxidants prevent oxidative processes by inhibiting the initiation or propagation of an oxidative chain reaction even when the amount of the antioxidant is less than the substance to be oxidized (3). During the last three decades the antioxidant-based drug formulations for the prevention and treatment of some oxidative stress related diseases have appeared. Reactive oxygen species can generate oxidative stress and play a role in the onset of nearly 150 pathophysiological disorders such as rheumatid arthritis, diabetes mellitus, inflammatory conditions, cancer, heart, genotoxicity diseases, early ageing (4). Several free radicals such as superoxide anion, hydrogen peroxide, nitric oxide, lipid peroxyl, singlet oxygen, lipid peroxide and hydroxyl radical, which are produced by all aerobic organisms and can easily react with most biological molecules including proteins, lipids, lipoproteins, and DNA have been reported in the literature (5). Therefore, algal species as alternative materials to extract natural antioxidative compounds have attracted much attention. There are epidemiological data supported by rodent model studies demonstrating protective effects of dietary kelps and other red and green algae against mammary, intestinal and skin carcinogenesis (6).

Various *In vitro* methods have been developed to analyze the potential of natural antioxidants such as pure compounds and plant extracts. *In vitro* methods can be divided into two major groups: 1) hydrogen atom transfer reactions such as Oxygen Radical Absorbance Capacity (ORAC), Total radical trapping antioxidant potential (TRAP) and β-carotene bleaching; 2) Electron transfer reactions like trolox equivalent antioxidant capacity (TEAC), Ferric reducing antioxidant power (FRAP), α-diphenyl-β-picryl-hydrazyl radical scavenging assay (DPPH), superoxide anion radical scavenging assay, hydroxyl radical scavenging assay, nitric oxide radical scavenging assay and total phenol assay have been reported in the literature (7). These methods are very popular and sensitive but to estimate antioxidant properties of plant materials, it is necessary to carry out more than one method since the phytochemicals are complex in nature (8). Many researchers have reported on the antioxidant and antimicrobial activity of seaweeds (9). However, reports on the antioxidant and antimicrobial activity of seaweed extracts from Mahabalipuram, Tamilnadu, India are very limited. The bioactive properties such as antioxidant, antimicrobial and antiproliferative effects of three seaweeds *Gracilaria corticata*, *Enteromorpha antenna*, *Enteromorpha linza* were analyzed using various *in vitro* assays. 

## 2. Objectives

The aim of this study was to investigate the bioactive properties of three seaweed samples; *Enteromorpha antenna*, *Enteromorpha linza* and *Gracilaria corticata* were collected from shorelines of Mahabalipuram, Tamilnadu.

## 3. Materials and Methods

### 3.1. Collection of Seaweeds

Three seaweed samples were collected along the Mahabalipuram shoreline, Tamil Nadu, and were identified as *Enteromorpha antenna*, *Gracilaria corticata* and *Enteromorpha linza*. Nine bacterial strains namely *Bacillus subtilis*, *Staphylococcus aureues*, *Micrococcus luteus*, *Escherichia coli*, *Vibrio cholerae*, *Shigella dysenteriae*, *Salmonella typhi*, *Klebsiella pneumoniae* and *Pseudomonas aeuroginosa* were obtained from Microbial Type culture collection & Gene Bank, (MTCC) Chandigarh, India. Two cancer cell lines namely MCF-7 (breast cancer cell line) and HepG2 (Liver cancer cell line) and VERO (normal cell line) were purchased from the National Centre for Cell Science (NCCS), Pune. All chemicals and solvents used were of highest purity grade.

### 3.2. Preparation of the Extracts

All samples were brought to the laboratory in plastic bags containing sea water to prevent evaporation. Few collected seaweeds were preserved for identification. Algae samples were cleaned such that epiphytes and necrotic parts were removed. Samples were rinsed with sterile water and shade dried for 7-14 days and ground thoroughly to powder in a kitchen-type blender. The extraction of the sample was carried out sequentially with different solvents of increasing polarity namely: chloroform, ethyl acetate, acetone, butanol, methanol, ethanol, and water by macerating the samples in the respective solvents (1:10, w/v) on a rotary shaker at 150 rpm at room temperature (25-30˚C) for 72 hours. The extracts from three consecutive soakings were pooled and filtered using filter paper (Whatmann No.4); the obtained filtrate was evaporated and the residues (crude extracts) obtained were suspended in the DMSO to a final concentration of 100 µg/mL; the extracts were stored at -20˚C (10).

### 3.3. Analysis of Antioxidant Activity

#### 3.3.1. Determination of Total Phenolics

The total soluble phenolic contents in seaweed were determined with Folin Ciocalteau reagent (11). To each of the seaweed extracts (100 µg/mL), 1 mL of Folin Ciocalteau reagent (1:10 v/v) was added and incubated at room temperature for 5 minutes. 1 mL of 7% sodium carbonate solution was added and incubated at room temperature for 90 minutes. The absorbance was measured at 720 nm using UV spectrophotometer (Shimadzu UV 1600). The same procedure was carried out for gallic acid (0.2-1 mg/mL) standard. The total phenol content of the extracts was obtained by using the standard curve. The total phenol content was expressed as gallic acid equivalent in %, w/w of the extracts.

#### 3.3.2. DPPH Free Radical Scavenging Assay 

Methanolic seaweed extracts and α-tocopherol (standard) were aliquated into series of concentrations (10-120 µg/mL). 1mL of freshly prepared 0.1 mM Methanolic DPPH solution was added and incubated in the dark for 20 minutes. The absorbance was measured at 517 nm. A similar procedure was repeated with distilled water instead of the extract, which served as a control while α tocopherol was used as a standard. All the tests were performed in triplicates. The percentage of free radical scavenging was calculated using the formula below (12, 13).

Free Radical Scavenging (%) = [(Control OD-Sample OD)/Control OD]/100

#### 3.3.3. Nitric Oxide Radical Scavenging Activity

Seaweed extract (100 µg/mL) was treated with 3 mL of 10 mM sodium nitroprusside in phosphate buffer. The resulting solution was then incubated at 25˚C for 150 minutes. From the above solution, 0.5 mL was taken and 1 mL of 0.33% Sulphanilic acid was added and incubated at room temperature for 5 minutes. 1 mL of 0.1% Napthylethylenediamine dihydrochloride was added and incubated at 25˚C for 30 minutes. The absorbance of pink chromophore formed during diazotization was determined by using a UV spectrophotometer at 546 nm. Blank solutions were prepared without adding sodium nitroprusside in the mixture. Experiments were repeated with distilled water without the plant extract, which acts as a control. All the tests were performed in triplicates and a standard graph was plotted by using L-ascorbic acid (10-100 μg/mL). The percentage of scavenging activity was calculated by using the standard graph (14).

#### 3.3.4. Hydrogen Peroxide Radical Scavenging Activity

Seaweed extract (100 µg/mL) was treated with 0.6 mL of 40 mM H_2_O_2_ solution prepared in phosphate buffer (7.4). After incubation at 37˚C for 10 minutes, absorbance was measured at 230 nm. Phosphate buffer was used as the corresponding blank solutions. A similar procedure was repeated with distilled water instead of the extract, which served as a control. While ascorbic acid (20–100 μg/mL) was used as a standard. A decrease in absorbance indicated an increase in free radical scavenging activity. The percentage of scavenging activity was calculated (15).

#### 3.3.5. Hydroxyl Radical Scavenging Activity

An assay mixture containing EDTA (1 mM), FeCl_3_ (10 mM), H_2_O_2_ (10 mM) and deoxyribose (10 mM) was added to the seaweed extracts (100 μg/mL) dissolved in distilled water with ascorbic acid (1 mM) in 50 mM phosphate buffer. The mixture was incubated at 37˚C for 1 hour and 1.0 mL of the incubated mixture was mixed with 1 mL of 10% TCA and 1 mL of 0.4% TBA (in glacial acetic acid, pH adjusted by NaOH) to develop the pink chromagen measured at 532 nm. BHT (20-100 μg/mL) was used as the positive control and the standard graph. The hydroxyl radical scavenging activity of the extract is reported as percentage inhibition of deoxyribose degradation and was calculated as previously reported (16).

#### 3.3.6. Reducing Power Activity

The seaweed extract (100 μg/mL) was mixed with 2.5 mL of 1% potassium ferricyanide and phosphate buffer (2.5 mL, pH = 6.6). The mixture was incubated at 50˚C for 20 minutes. 2.5 mL of 10% trichloroacetic acid was added to the mixture, then centrifuged at 3000 x g for 10 minutes. The upper layer of the solution (2.5 mL) was mixed with 2.5 mL of distilled water and 0.5 mL of freshly prepared 0.1% ferric chloride solution. An increase in absorbance due to the reducing power activity of extracts was determined at 700 nm using the blank solution containing the above-mentioned solution without ferric chloride. The same procedure was repeated with distilled water in place of the extract and served as a control. The activity of extracts was compared with ascorbic acid (10-100 µg/mL) used as a standard (17).

#### 3.3.7. FRAP Assay

Seaweed extracts (100 µg/mL) were mixed with 1.5 mL of freshly prepared FRAP reagent (25 mL of 300 mM/L of acetate buffer pH 3.6, 2.5 mL of 10 mM/L 2, 4, 6 tripyridyl S triazine (TPTZ) in 40 mM/L of HCl, 20 mM/L of ferric chloride solution). The absorbance was measured at 593 nm during the zeroth minute after vortexing. Thereafter, samples were placed at 37˚C in a water bath and absorption was again measured after 4 minutes. The results are expressed as mM of FRAP per liter and were estimated using aqueous FeSO_4_. 7H_2_O (200–1000 mM) as a standard for calibration. The relative activity of the sample was compared with standard L-ascorbic acid (2–10 μg/mL). The absorbance was measured at 593 nm. Linear regression curve was plotted for the standard and FRAP values (mM of Fe (II) per Liter) were calculated from the regression equation (18).

### 3.4. Antimicrobial Assay

The microorganisms were inoculated in 5 mL of nutrient broth and incubated for 24 hours in a shaker incubator at 37˚C. After 24 hours they were re-inoculated and grown for 4 hours and then used for swabbing. Methanolic seaweed extracts (10 mg/mL) stock was prepared in DMSO from which 250 µg, 500 µg, 750 µg and 1 mg were sequentially checked for activity. The antimicrobial activity was evaluated using the well diffusion method. Wells were punched using a sterile 0.6 cm cork borer in nutrient agar plates swabbed with the test microorganism. For each microorganism, negative control DMSO was loaded instead of the extract and the plates were incubated at 37˚C for 24 hours (19). The inhibition zone diameter for each well in millimeter was compared with the positive control streptomycin. The experiments were carried out in triplicates.

### 3.5. Analysis of Anti Proliferative Effect Elicited by Methanolic Crude Extract 

The 48 hour monolayer culture of the cells at a concentration of one lakh cells per well were seeded into 24 well titer plates. The plates were microscopically examined for confluent monolayers. The MEM was removed without disturbing the cell sheet and monolayer of cells was washed twice with MEM without FCS to remove the dead cells and excess FCS. To the washed cell sheet, 1 mL of medium without FCS containing defined concentration of the seaweed extract was added with a dilution range of 1:1 to 1:64. 1 mL MEM without FCS was used as a control. The plates were incubated in a 5% CO_2_ incubator and observed for cytotoxicity using inverted microscope at 20x objective. The medium from the wells was removed carefully for the MTT assay. Each well was washed with MEM without FCS, 2–3 times and 200 μL of MTT concentrate (5 mg/mL) was added and incubated for 6-7hrs in 5% CO_2_ incubator. After incubation 1mL of DMSO was added to each well and mixed by pipetting and was left for 45 seconds. The OD values were read at 595 nm using a spectrophotometer, having DMSO as a blank. A graph was plotted with concentration of the drug versus relative cell viability. The cell viability was calculated using the formula below (20, 21):

Cell Viability, % = (Mean OD/Control OD) x 100

## 4. Results

### 4.1. Total Phenolic Content

The total phenolic content of the seaweed extracts was measured by the Folin-Ciocalteau method and expressed as gallic acid equivalents (GAE). The total phenolic content was highest for methanolic extract of *E. antenna* (1.816 ± 0.05 GAE mg/g), *G. corticata* (1.509 ± 0.023 GAE mg/g), and *E. linza* (0.912 ± 0.032 GAE mg/g) compared to the other extracts.

### 4.2. DPPH Radical Scavenging Activity

The free radical scavenging activity of methanolic extract of seaweed was assessed by the DPPH assay (Table 1). A significant decrease in the concentration of DPPH radical was observed due to the scavenging ability of the seaweeds. Tocopherol was used as a standard. The result showed that the IC_50_ values for extract of *E. antenna*, *G. corticata*, *E. linza*and standard were 70 μg/mL, 72.9 μg/mL, 110 μg/mL, and 80 μg/mL respectively. This indicates they are good sources of natural antioxidants. 

**Table 1.** DPPH Radical scavenging Activity

Concentration of Methanolic Extracts, µg/mL	Radical Scavenging, %			
	*E. antenna*	*G. corticata*	*E. linza*	α-tocopherol
**10**	3	11	7	10
**20**	12	22	17	18
**30**	24	28	19	24
**40**	30	35	20	35
**50**	41	38	28	43
**60**	46	40	30	47
**70**	52	48	36	49
**80**	57	53	38	50
**90**	60	60	39	53
**100**	62	72	43	55
**110**	64	-	50	57
**120**	67	-	55	59

### 4.3. Hydrogen Peroxide Radical Scavenging Activity

The hydroxyl radicals produced by hydrogen peroxide were scavenged by the plant extracts and showed a decrease in absorbance due to the reduction of these radicals at 230 nm. The methanolic extract (100 µg/mL) of *E. antenna, G. corticata*, and *E. linza *had hydrogen peroxide scavenging activity of 87%, 79% and 53%, which was observed to be higher than the other extracts. However ascorbic acid standard had 89% scavenging activity (Tables 2, 3 and 4). 

**Table 2.** Antioxidant Activity of *E. antenna* Samples

Seaweed Extract, 100 µg/mL	Total Polyphenolic Content, GAE mg/g	Scavenging of Radicals, %			Reducing Power	FRAP, mM of Fe(II)/L
		HO^•^	NO^•^	H_2_O_2_		
**Chloroform, Mean ± SD**	0.232 ± 0.031	20 ± 0.06	32.25 ± 0.14	25 ± 0.15	0.502 ± 0.04	125 ± 0.22
**Ethyl acetate, Mean ± SD**	0.463 ± 0.04	40 ± 0.02	28.5 ± 0.12	30.2 ± 0.10	0.394 ± 0.03	620 ± 0.17
**Acetone, Mean ± SD**	0.834 ± 0.08	36 ± 0.04	52 ± 0.06	58 ± 0.45	0.65 ± 0.01	322.3 ± 0.26
**1-butanol, Mean ± SD**	0.423 ± 0.014	11 ± 0.05	4.01 ± 0.12	47 ± 0.53	0.457 ± 0.02	190.85 ± 0.5
**Methanol, Mean ± SD**	1.816 ± 0.05	74 ± 0.08	77 ± 0.09	87.6 ± 0.26	1.334 ± 0.03	750 ± 0.08
**Ethanol, Mean ± SD**	0.567 ± 0.21	56 ± 0.023	42.15 ± 0.15	66 ± 0.57	0.533 ± 0.02	525 ± 0.04
**Water, Mean ± SD**	0.328 ± 0.03	48 ± 0.04	23 ± 0.11	37.5 ± 0.34	0.438 ± 0.05	385.51 ± 0.21

**Table 3.** Antioxidant Activity of *G. corticata *Samples

Seaweed Extract, 100 µg/mL	Total Polyphenolic Content, GAE mg/g	Scavenging of Radicals, %			Reducing Power	FRAP, mM of Fe(II)/L
		HO^•^	NO^•^	H_2_O_2_		
**Chloroform, Mean ± SD**	0.345 ± 0.011	16.42 ± 0.02	28.45 ± 0.04	22.28 ± 0.93	0.375 ± 0.05	100.15 ± 0.02
**Ethyl acetate, Mean ± SD**	0.597 ± 0.083	38.12 ± 0.03	34.02 ± 0.07	37.85 ± 0.59	0.61 ± 0.021	201.06 ± 0.13
**Acetone, Mean ± SD**	0.664 ± 0.072	50.37 ± 0.12	18.16 ± 0.24	58.93 ± 0.67	0.577 ± 0.05	285.61 ± 0.16
**1-butanol, Mean ± SD**	0.284 ± 0.089	13.55 ± 0.01	5.65 ± 0.03	36.44 ± 0.50	0.231 ± 0.09	160.03 ± 0.07
**Methanol, Mean ± SD**	1.509 ± 0.026	72.20 ± 0.02	68.23 ± 0.09	79.02 ± 0.33	1.3025 ± 0.04	625.25 ± 0.22
**Ethanol, Mean ± SD**	0.440 ± 0.054	20.99 ± 0.06	40.36 ± 0.18	30.57 ± 0.46	0.319 ± 0.04	543.21 ± 0.32
**Water, Mean ± SD**	0.565 ± 0.059	26.95 ± 0.06	48.42 ± 0.03	34.69 ± 0.57	0.619 ± 0.03	330.01 ± 0.18

**Table 4.** Antioxidant Activity of *E. linza *Samples

Seaweed Extract, 100 µg/mL	Total Polyphenolic Content, GAE mg/g	Scavenging of Radicals, %			Reducing Power	FRAP, mM of Fe(II)/L
		HO^•^	NO^•^	H_2_O_2_		
**Chloroform, Mean ± SD**	0.230 ± 0.014	12.96 ± 0.04	14.32 ± 0.13	11.23 ± 0.40	0.401 ± 0.05	75.00 ± 0.32
**Ethyl acetate, Mean ± SD**	0.377 ± 0.021	20±0.013	10.12±0.10	22.46 ± 0.54	0.451 ± 0.01	341.16 ± 0.26
**Acetone, Mean ± SD**	0.560 ± 0.012	26.12 ± 0.01	20.58 ± 0.03	38.03 ± 0.65	0.499 ± 0.01	140.07 ± 0.16
**1-butanol, Mean ± SD**	0.156 ± 0.025	8 ± 0.08	2.68 ± 0.18	14.26 ± 0.63	0.259 ± 0.03	85.01 ± 0.02
**Methanol, Mean ± SD**	0.912 ± 0.032	62.6 ± 0.03	31.46 ± 0.06	52.78 ± 0.417	0.766 ± 0.05	400.00 ± 0.02
**Ethanol, Mean ± SD**	0.263 ± 0.046	18 ± 0.02	21.3 ± 0.23	31.2 ± 0.60	0.342 ± 0.02	311.00 ± 0.08
**Water, Mean ± SD**	0.242 ± 0.058	16 ± 0.015	18.12 ± 0.12	26.6 ± 0.32	0.532 ± 0.04	219.06 ± 0.12

### 4.4. Hydroxyl Radical Scavenging Ability

Hydroxyl radical scavenging ability of methanolic extract of *E. antenna*, *G. corticata*, *E. linza*, and BHT at 100 µg/mL was observed to be 74%, 72%, 62%, and 71% respectively (Tables 2, 3 and 4). 

### 4.5. Nitric Oxide Radical Scavenging Activity

Suppression of NO• release may be attributed to a direct NO• scavenging effect as all the seaweed extracts decreased the amount of nitrite generated from the degradation of sodium nitroprusside *in vitro*. In this method Nitroprusside in aqueous solution at the physiological pH produces Nitric oxide and it reacts with molecular oxygen to form nitrite ions. The antioxidant scavenges the production of Nitric oxide. Nitric oxide scavenging ability of *E. antenna*, *G. corticata *and *E. linza *was 77%, 68% and 31%, which was observed to be lower than ascorbic acid which had radical scavenging activity of 87% (Tables 2, 3 and 4). 

### 4.6. Reducing Power

Reducing power is associated with antioxidant activity and may serve as a significant reflection of the antioxidant activity (22). Compounds with reducing power indicate that they are electron donors and can reduce the oxidized intermediates of lipid peroxidation processes, so that they can act as primary and secondary antioxidants (23). Electron donating ability of the seaweed extracts and thus their potential role in reduction of the oxidized intermediates of lipid peroxidation process was determined. The reducing capacity of various extracts (100 µg/mL) was compared with the ascorbic acid standard (10-100 µg/mL). Reducing capacity of methanolic extract of *E. antenna *and *G. corticata *was equivalent to the standard, ascorbic acid (100 µg/mL). *E. antenna *and *G. corticata *can be considered as a potent source of natural antioxidants as it acts as a good indicator of its potential antioxidant property (Tables 2, 3 and 4) (24). 

### 4.7. FRAP Assay

The reducing capacity of the samples were analyzed by the FRAP method measuring the absorbance at 593 nm and antioxidant power was calculated. The FRAP values were found to be highest for methanolic extracts of *G. corticata *(625 FRAP units), The FRAP values were found to be highest for methanolic extracts of *E. antenna *(750 units), *G. corticata *(625 units) and *E. linza *(400 units) respectively. The antioxidant profile for *G. corticata *, *E. linza *, *E. antenna *were tabulated in (Tables 2, 3 and 4). 

### 4.8. Correlation Analysis

**Figure 1.**
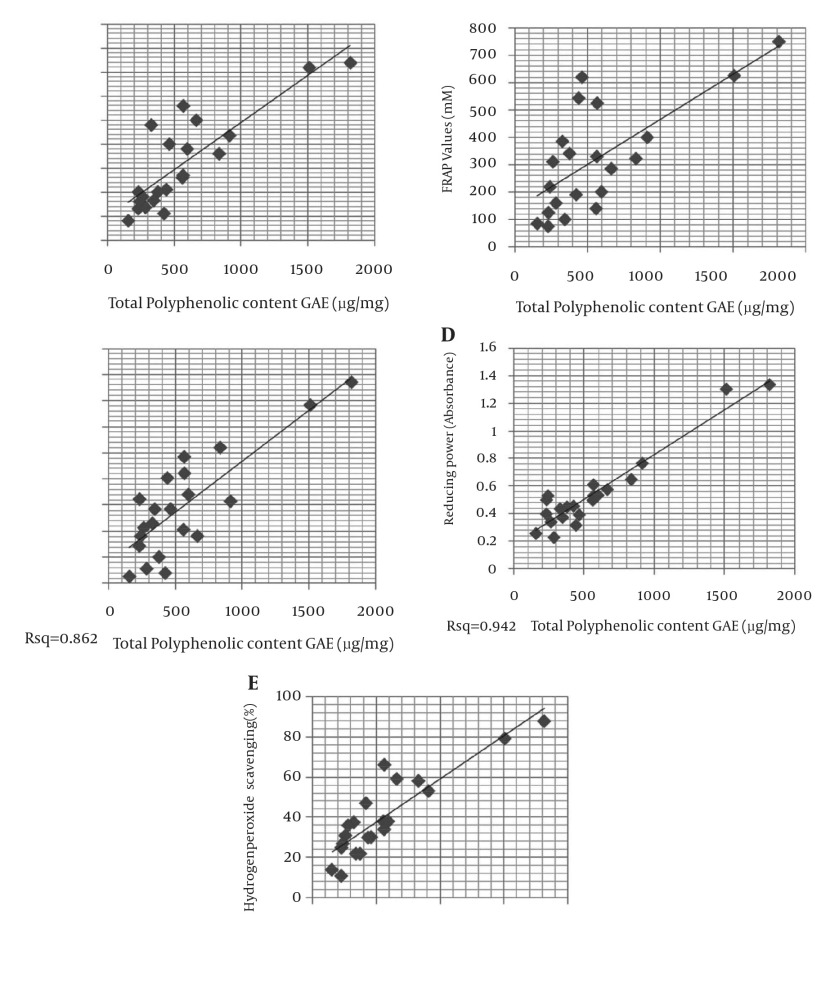
Correlation Between the Contents of Total Phenols in Seaweeds and Their Antioxidant Capacity as Determined by A. Antioxidant Assay using Hydroxyl Radical Method, B. FRAP Method, C. Nitric Oxide Method, D. Reducing Power Method and E. Hydrogen Peroxide Method

The antioxidant property of various seaweeds such as edible brown, green and red seaweeds has been correlated to their phenolic content (25). From the results obtained, it was clear that the correlation between total phenolics and total reducing power was highest (R^2^ = 0.942) and lowest when correlated with respective FRAP values (R^2^ = 0.707). The correlation graphs have been represented in Figure 1. 

### 4.9. Antimicrobial Activity

The antibacterial activities of three seaweeds were assessed against nine food borne pathogens using a disc diffusion assay. The antimicrobial activities were considered to be an indicator of the capacity of seaweeds to synthesize bioactive secondary metabolites. The methanolic extracts showed average zones of inhibition in all tested microorganisms except *Pseudomonas aeruginosa*, in comparison to the positive control, streptomycin (Table 5).

**Table 5.** Antimicrobial Activity of Crude Methanolic Seaweed Extracts

Microbial Strains	Zone of Inhibition, mm											
	Conc of *E. antenna*, µg/mL				Conc of *G. corticata*, µg/mL				Conc of *E. linza*, µg/mL			
	250	500	750	1000	250	500	750	1000	250	500	750	1000
***Salmonella typhi***	12	14	15	18	-	-	11	13	10	11	12	13
***Vibrio cholerae***	10	11	13	16	10	12	15	19	14	15	17	18
***Klebsiella pneumoniae***	11	14	16	21	12	14	17	22	11	12	15	17
***Escherichia coli***	10	12	14	15	12	14	15	18	12	15	18	20
***Shigelladysenteriae***	10	11	13	15	-	-	12	14	11	12	15	17
***Staphylococcus aureues***	10	13	15	17	-	10	12	14	10	11	14	18
***Bacillus subtilis***	10	11	12	14	-	-	12	15	-	-	11	14
***Micrococcus luteus***	13	14	15	17	10	11	13	14	12	13	15	16
***Pseudomonas aeuroginosa***	-	-	-	-	-	-	-	-	-	-	-	-

### 4.10. Anti-proliferative Activity

**Figure 2.**
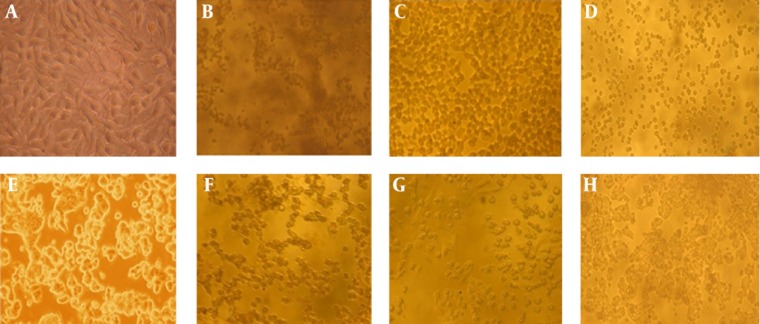
Anti-Proliferative Activity of *E. antenna*, *G. corticata* and *E. linza* on HepG2 Cells and MCF7 Cells A. Normal HepG2, B. *E. antenna* high toxic, C. *G. corticata* high toxic, D. *E. linza high* toxic, E. Normal MCF7, F. *E. antenna* high toxic, G. *G.corticata* high toxic, H. *E. linza* high toxic.

The anti proliferative activity on cancer cell lines HepG2, MCF7 and normal VERO cell line were determined using the MTT cytotoxicity assay for the crude methanol extracts of all the three samples at various concentrations. *G. corticata* (91.44%) showed highest toxic effect on HepG2 cells followed by *E. antenna* (87.43%) and *E. linza* (71.44%). Similarly *G. corticata* (93.75%) also showed highest toxic effect on MCF7 cells followed by *E. linza* (84.77%) and *E. antenna* (64.75%) (Figure 2). Only at a high concentration of 1 mg/mL, 60% cytoxicity was seen in normal VERO cell lines.

## 5. Discussion

The usage of seaweeds by the Indian population is very much limited. In India seaweeds are mainly used for in food products as functional ingredient such as agar, alginate etc. Addition of such food products improves not only functional and sensory properties of food but also the nutritional quality due to the presence of many bioactive components like antioxidative substances (26). Due to the variability within the species growing conditions, time of maturity, climatic and environmental conditions, the bioactivities and chemical compositions in sea weeds will also vary (27). The byproducts of the metabolic process on exposure to the radiation produce free radicals, which are highly reactive molecules with an unpaired electron. The chain reaction initiated by the free radicals induces the disintegration of cell membranes and its cell components. Also, the free radicals are the major cause of food deterioration through oxidation of lipids, which ultimately affects the palatability of foods. The antioxidants have main roles in scavenging the free radicals, maintaining the cell integrity, slow down ageing and prevent the development of complications associated with oxidative stress- related diseases and cancer (28). Hence, intervention of a novel antioxidant may have a therapeutic effect and also maintain the freshness of food products. Natural antioxidants were found to be a better alternate to synthetic antioxidants since it exhibits adverse effects. 

DPPH is considered as a good kinetic model for peroxyl radicals (29). The decrease in absorbance at 517 nm shows the ability of seaweeds to scavenge the DPPH radicals. The results of the DPPH scavenging activity assay for the extracts of all the seaweeds exhibits good results. *G. corticata* was found to be exhibiting significantly higher DPPH scavenging activity (72%) than *E. antenna* species (62%) and *E. linza* (43%) when compared with standard tocopherol (55%). OH• has a short half-life and is the most reactive, known to be capable of abstracting hydrogen atoms from cell membranes and they bring about peroxidic reactions of lipids (30). The prevention of 2-deoxyribose-2-ribose degradation facilitated by the mixture of seaweed extract and BHT (100 μg/mL) and removed the hydroxyl radicals. Among the three seaweeds under investigation, *E. antenna* exhibited the maximum scavenging effect of OH• (74%).

Nitric oxide is a free radical that is generated when sodium nitroprusside reacts with oxygen to form nitrite, induces the inflammatory response and its toxicity multiplies if it reacts with O2• radicals to form peroxynitrite (31). The present results suggest that *E. antenna* and *G. corticata* might be potent and novel therapeutic agents for scavenging of NO and the regulation of pathological conditions caused by excessive generation of NO and peroxynitrite. 

The highly reactive hydroxyl radical (•OH) generated via a biologic Fenton reaction (hydrogen peroxide with Fe^2+^ and Cu^2+^ causes cytotoxic effect through the alteration of [Ca^2+^] homeostasis (32). The results suggested that *E. antenna* and *G. corticata* expressed hydrogen peroxide scavenging activity of 7.60% and 79.02% and can be a good resource of antioxidants for removing H_2_O_2_ and thus protecting food systems. The reducing ability was found highest for *E. antenna* followed by *G. corticata* and *E. linza,* respectively. Compounds with reducing power indicate that they are electron donors and can act as primary and secondary antioxidants by reducing the oxidized intermediates of lipid peroxidation (23). The presence of reductones in a compound indicates its greater reducing ability, which shows antioxidative potential by breaking the free radical chain by donating a hydrogen atom (17). 

The results of the antioxidant assays indicated that the methanolic extracts of *E. antenna* and *G. coticata* are the best source of antioxidant compounds among the seaweeds investigated. It has been reported that sun drying and subsequent storage of algae will considerably decrease the levels of these labile antioxidants such as L- ascorbate (33). A requirement for endogenous antioxidant capacity in algae is implicit, due to the fact that algae, as intertidal organisms, require protection against UV irradiation (34). The antibacterial activity in algae have been variously reported as bromophenols, carbonyls, halogenated aliphatic compounds, terpenes, isoprenylated and brominated hydroquinones, as well as phlorotannins. The antiproliferative activity of crude extracts might be related to their ability to scavenge free radicals and carcinogenic agents. Considering their great taxonomic diversity, investigations related to the search of new antioxidative compounds from algae can be seen as an almost unlimited field.

## References

[1] Sinnathambi A, Mazumder PM, Ashok P, Narayanan LS (2008). In Vitro Antioxidant and Free Radical Scavenging Activity of Alstonia scholaris Linn. R. Br.. Iran J Pharmacolo Ther..

[2] Moreau J, Pesando D, Bernard P, Caram B, Pionnat JC (1988). Seasonal variations in the production of antifungal substances by some dictyotales (brown algae) from the French Mediterranean coast.. Hydrobiologia..

[3] Pham-Huy LA, He H, Pham-Huy C (2008). Free radicals, antioxidants in disease and health.. Int J Biomed Sci..

[4] Kalim MD, Bhattacharyya D, Banerjee A, Chattopadhyay S (2010). Oxidative DNA damage preventive activity and antioxidant potential of plants used in Unani system of medicine.. BMC Complement Altern Med..

[5] Cui H, Kong Y, Zhang H (2012). Oxidative stress, mitochondrial dysfunction, and aging.. J Signal Transduct..

[6] Yuan YV, Walsh NA (2006). Antioxidant and antiproliferative activities of extracts from a variety of edible seaweeds.. Food Chem Toxicol..

[7] Huang D, Ou B, Prior RL (2005). The chemistry behind antioxidant capacity assays.. J Agric Food Chem..

[8] Salazar R, Pozos ME, Cordero P, Perez J, Salinas MC, Waksman N (2008). Determination of the antioxidant activity of plants from Northeast Mexico.. Pharm Biol..

[9] Plaza M, Cifuentes A, Ibáñez E (2008). In the search of new functional food ingredients from algae.. Trends Food Sci Technol..

[10] Devi KP, Suganthy N, Kesika P, Pandian SK (2008). Bioprotective properties of seaweeds: in vitro evaluation of antioxidant activity and antimicrobial activity against food borne bacteria in relation to polyphenolic content.. BMC Complement Altern Med..

[11] Singleton VL, Rossi JA (1965). Colorimetry of total phenolics with phosphomolybdic-phosphotungstic acid reagents.. Am J Enol Viticult..

[12] Stanislav B, Marianna K, Klaudia J, Attila G, Marian V (2012). Free radical scavengering capacity of Papaver somniferum L. and detection of pharmacologcically active alkaloids using capillary electrophoresis.. J Microbiol Biotechnol Food Sci..

[13] Badami S, Prakash OM, Dongre SH, Suresh B (2005). In vitro antioxidant properties of Solanum pseudocapsicum leaf extracts.. Indian J Pharmacol..

[14] Vadnere GP, Patil AV, Wagh SS, Jain SK (2012). In vitro free radical scavenging and antioxidant activity of Cicer arietinum L.(Fabaceae).. Int J PharmTech Res..

[15] Ebrahimzadeh MA, Nabavi SM, Nabavi SF, Bahramian F, Bekhradnia AR (2010). Antioxidant and free radical scavenging activity of H. officinalis L. var. angustifolius, V. odorata, B. hyrcana and C. speciosum.. Pak J Pharm Sci..

[16] Mandal P, Misra TK, Ghosal M (2009). Free-radical scavenging activity and phytochemical analysis in the leaf and stem of Drymaria diandra Blume.. Int J Integr Biol..

[17] Sikder AA, Rahman A, Islam R, Kaisar A, Rahman MS, Rashid MA (2010). In vitro antioxidant, reducing power, free radical scavenging and membrane stabilizing activities of Spilanthes calva. Bangladesh Pharm.. Bangladesh pharm J..

[18] Benzie IF, Strain JJ (1996). The ferric reducing ability of plasma (FRAP) as a measure of "antioxidant power": the FRAP assay.. Anal Biochem..

[19] Oloke EA, Ayandele AA, Adegunlola CO (2012). Phytochemical, antioxidant and antimicrobial assay of mushroom metabolite from Pleurotus pulmonarius –LAU 09 (JF736658).. J Microbiol Biotech Res..

[20] Ng LT, Wu SJ (2011). Antiproliferative Activity of Cinnamomum cassia Constituents and Effects of Pifithrin-Alpha on Their Apoptotic Signaling Pathways in Hep G2 Cells.. Evid Based Complement Alternat Med..

[21] Predes FS, Ruiz AL, Carvalho JE, Foglio MA, Dolder H (2011). Antioxidative and in vitro antiproliferative activity of Arctium lappa root extracts.. BMC Complement Altern Med..

[22] Oktay M, Gülçin İ, Küfrevioğlu Öİ (2003). Determination of in vitro antioxidant activity of fennel (Foeniculum vulgare) seed extracts.. LWT-Food Sci Technol..

[23] Jayanthi P, Lalitha P (2011). Reducing power of the solvent extracts of Eichhornia crassipes (Mart.) Solms.. Int J Pharm Pharm Sci..

[24] Arulpriya P, Lalitha P, Hemalatha S (2010). Invitro antioxidant testing of the extracts of Samanea saman (Jacq.) Merr.. Der Chemica Sinica..

[25] Ganesan P, Kumar CS, Bhaskar N (2008). Antioxidant properties of methanol extract and its solvent fractions obtained from selected Indian red seaweeds.. Bioresour Technol..

[26] Mamatha BS, Namitha KK, Senthil A, Smitha J, Ravishankar GA (2007). Studies on use of Enteromorpha in snack food.. Food Chem..

[27] Manivannan K, Thirumaran G, Devi GK, Hemalatha A, Anantharaman P (2008). Biochemical composition of seaweeds from Mandapam coastal regions along Southeast Coast of India.. Am Eurasian J Botany..

[28] Wu XJ, Hansen C (2008). Antioxidant capacity, phenolic content, and polysaccharide content of Lentinus edodes grown in whey permeate-based submerged culture.. J Food Sci..

[29] Rackova L, Oblozinsky M, Kostalova D, Kettmann V, Bezakova L (2007). Free radical scavenging activity and lipoxygenase inhibition of Mahonia aquifolium extract and isoquinoline alkaloids.. J Inflamm (Lond)..

[30] Vladimir-Knezevic S, Blazekovic B, Stefan MB, Alegro A, Koszegi T, Petrik J (2011). Antioxidant activities and polyphenolic contents of three selected Micromeria species from Croatia.. Molecules..

[31] Stadler K (2011). Peroxynitrite-driven mechanisms in diabetes and insulin resistance - the latest advances.. Curr Med Chem..

[32] Hazarika N, Singh P, Hussain A, Das S (2012). Phenolics content and antioxidant activity of crude extract of Oldenlandia corymbosa and Bryophyllum pinnatum.. Res J Pharm Biol Chem Sci..

[33] Cornish ML, Garbary DJ (2010). Antioxidants from macroalgae: potential applications in human health and nutrition.. Algae..

[34] Swanson AK, Druehl LD (2002). Induction, exudation and the UV protective role of kelp phlorotannins.. Aquat Bot..

